# Clinical, Radiological, and Pathological Diagnosis of Fibro-Osseous Lesions of the Oral and Maxillofacial Region: A Retrospective Study

**DOI:** 10.3390/diagnostics12020238

**Published:** 2022-01-19

**Authors:** Ellen Pick, Tobias Schäfer, Adib Al-Haj Husain, Niels J. Rupp, Lukas Hingsammer, Silvio Valdec

**Affiliations:** 1 Clinic of Cranio-Maxillofacial and Oral Surgery, Center of Dental Medicine, University of Zurich, 8032 Zurich, Switzerland; ellen.pick@zzm.uzh.ch (E.P.); tobias.schaefer@uzh.ch (T.S.); adib.alhaj@gmail.com (A.A.-H.H.); 2 Department of Pathology and Molecular Pathology, University Hospital Zurich, University of Zurich, 8091 Zurich, Switzerland; niels.rupp@usz.ch; 3 Department of Oral and Maxillofacial Surgery, Medical University of Vienna, 1090 Vienna, Austria; mail@drhingsammer.at; 4 Department of Stomatology, Division of Periodontology, Dental School, University of São Paulo, Butantã 2227, SP, Brazil

**Keywords:** fibro-osseous lesions, benign tumor, fibrous dysplasia, ossifying fibroma, cemento-osseous dysplasia, radiology, histology, oral anatomy, demographics

## Abstract

Background: Fibro-osseous lesions (FOL) of the jaw represent a rare, benign group of lesions that share similar clinical, radiological, and histopathological features and are characterized by progressive, variable replacement of healthy bone tissue by fibrous connective tissue. Methods: This retrospective study aimed to evaluate the incidence of fibro-osseous lesions and to reassess the efficacy of case-specific treatment management from a clinical, radiological, and histopathological perspective based on 14 years of data. Results: Forty-four patients with a radiological and/or histopathological diagnosis of benign FOLs were identified and re-evaluated. Cemento-osseous dysplasia was the most common group of FOLs present in our patient cohort (45%), followed by ossifying fibroma (39%) and fibrous dysplasia (16%). The diagnostic imaging technique of choice was CBCT (68%), followed by PAN (18%), with most patients (95 %) additionally undergoing biopsy. The mean age of the patients at the time of diagnosis was 40.54 ± 13.7 years, with most lesions being located in the mandible (86%), with females being predominantly affected (73%). Conclusion: An interdisciplinary approach that analyzes all case-specific factors, including demographic data, medical history, intraoperative findings, and, most importantly, histopathological and radiological features, is essential for an accurate diagnosis and key to avoiding inappropriate treatment.

## 1. Introduction

Fibro-osseous lesions (FOL) of the oral and maxillofacial region represent a rare, benign group of lesions that share similar clinical, radiological, and histopathological features. They are characterized by progressive, variable replacement of healthy bone tissue in the jaw by fibrous connective tissue containing varying amounts of mineralized substances that include bone, osteoid, and cementum-like material [1,2]. According to the latest 2017 WHO classification for odontogenic and maxillofacial bone tumors, other lesions such as cysts and specifically FOLs were reintroduced as a new lesion group in addition to the already implemented tumors [3]. Three main groups of FOLs can be distinguished: (1) fibrous dysplasia (FD); (2) cemento-ossifying fibroma (COF); and (3) cemento-osseous dysplasia (COD). In addition, familial gigantiform cementoma (FGC), a rare autosomal disease of unclear etiopathogenesis with familial clustering and multifaceted radiological characterization, and osteochondromatous lesions were added to the classification [4].

Due to the rarity and diverse presentation of FOLs, their diagnosis is challenging in routine clinical practice, especially since histopathological analysis, although essential for an accurate diagnosis, is not always sufficient. Because of the clinical inconspicuousness and lack of symptoms, the diagnosis of FOLs is often the result of an incidental imaging finding [5]. Clinically, a close positional relationship of the FOLs to one or more tooth roots is typical, therefore the affected teeth are usually vital [6]. Because inadequate surgical interventions may cause complications such as recurrence or progression of the lesion [7], a multimodality diagnostic approach requiring accurate indication-specific radiologic assessment by panoramic radiography (PAN) or cone-beam computed tomography (CBCT) and the surgeon’s clinical experience are fundamental for decision-making leading to optimized, personalized therapeutic planning. 

PAN is considered a primary and sufficient diagnostic tool for most FOLs, although in some atypical and indistinguishable cases, the need for three-dimensional information about the region of interest can justify the use of additional imaging such as CBCT or computed tomography (CT) [8]. From a radiological point of view, previous reports have shown that most fibrous dysplasia cases had specific radiographic features, such as ground-glass appearance, with about one-fifth having a mixed radiolucent and radiopaque appearance, whereas the majority of ossifying fibroma (OF) cases had unilocular radiolucency or a mixed radiolucent and radiopaque appearance [9]. Other imaging modalities of interest for detecting and monitoring bony lesions in the jaw, especially in repeated radiographic examinations, are magnetic resonance imaging [10] and ultrasound [11]. Ultrasound per se offers better detectability of bony lesions compared to conventional radiation-based imaging modalities. However, evidence-based information on its reliability and practicability in clinical routine is still lacking [12]. Histopathologically, the differential diagnosis still presents difficulties due to morphological overlaps, unless characteristic mutational analysis of the alpha subunit of the G protein gene (GNAS) reveals mutation and therefore leads to the diagnosis of fibrous dysplasia [13,14].

Within each group of FOLs, the treatment strategies can vary from simple periodic follow-up to a more invasive approach, including major resective surgeries of the affected jaw [15,16]. For example, COD and its subgroups are mainly diagnosed with a clinical and radiological examination, a biopsy is rarely required [5]. In contrast, ossifying fibroma and fibrous dysplasia are often excised as a treatment procedure, so conventional histomorphological analysis is used to confirm the initial clinical and radiological diagnosis [1,15,17]. However, difficulty arises with lesions that present atypically, in these cases treatment planning should always be based on a correlation between modality-oriented and case-specific clinical, radiologic, and histopathologic evidence. Although FOLs are considered benign tumors of the oral and maxillofacial region, some of these lesions exhibit an aggressive growth tendency, such as the juvenile ossifying fibromas (juvenile trabecular ossifying fibroma (JTOF) and juvenile psammomatoid ossifying fibroma (JPOF)), which may be associated with rapid and extensive bone expansion [18]. Fibrous dysplasia also has a low potential for malignant transformation, with an estimated risk of 0.4–6.7% [1]. Previous studies suggest that this malignant transformation may occur decades after initial diagnosis [18].

Due to the rarity and heterogeneous presentation of FOLs, there is no consensus on treatment and follow-up protocols in the current literature. Therefore, this retrospective study aimed to evaluate the incidence of fibro-osseous lesions and to reassess the efficacy of case-specific treatment management from a clinical, radiological, and histopathological perspective based on 14 years of data. To improve the quality of care, thorough knowledge of the diseases’ manifestations should be implemented to develop optimized decision-making in clinical and radiological workflows, covering the entire spectrum from the need for potential surgical intervention to radiological follow-up only.

## 2. Materials and Methods

For this retrospective study, all clinical, radiological, and laboratory reports of patients with a radiological and/or histopathological diagnosis of benign fibro-osseous lesions of the jaw treated at the Clinic of Cranio-Maxillofacial and Oral Surgery at the Center of Dental Medicine (University of Zurich) and the University Hospital of Zurich (University of Zurich) in Zurich, Switzerland between 2005 and 2019 were included in the study sample. Database searches were performed by two principal investigators (E.P., T.S.) in the electronic medical management systems VitoDent (Vitodata AG, Oberohringen, Switzerland) and KISIM (Cistec AG, Zurich, Switzerland), the local Picture Archiving and Communication System (PACS) (IMPAX EE R20, release XV, Agfa Healthcare, Mortsel, Belgium), and the database of the Department of Pathology (PathoPro Software, Institute of Medical Software, Saarbrücken, Germany). Patients who explicitly refused to participate in the study and patients with inadequate documentation in the medical record were excluded.

The overall incidence of fibro-osseous lesions and the distribution of their different types and subgroups, according to the WHO classification of 2017, were assessed. In addition, parameters such as age and gender of affected patients, imaging modality used for diagnosis (PAN, CT, CBCT), presence/absence of histopathological examination, use of resective surgery in which the entire lesion was removed after biopsy as further treatment and why this procedure was indicated, duration of postoperative recall-interval, postoperative complications, and recurrence or malignant transformation of the lesions were evaluated.

The data were analyzed using descriptive statistics; metric variables with means, standard deviations (SD), medians, interquartile ranges, minimums, and maximums and categorical variables with frequencies and percentages. The statistical analysis of the data was performed using IBM SPSS Statistics software (version 25.0, IBM Corp., Armonk, NY, USA).

The project (BASEC-Nr. 2018-0214) received ethical approval from the Cantonal Ethics Commission of Zurich, Switzerland (“Kantonale Ethikkommission Zürich”). Additionally, this retrospective study was performed in accordance with the 1964 Helsinki Declaration and its later revised ethical standards. The authors obtained permission from each study participant to reuse all health-related clinical data, radiological data, and biological specimens for research.

## 3. Results

### 3.1. Overview

**Figure 1:**
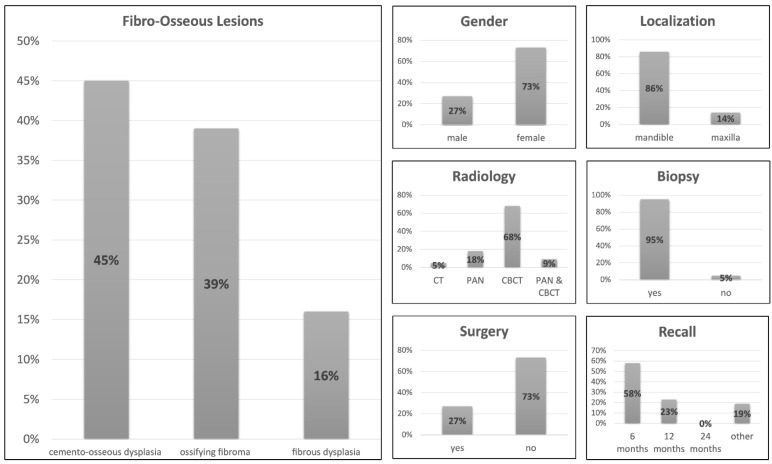
Overview: Parameters for all fibro-osseous lesions.

Radiological and histopathological reports were analyzed in 1207 patients in all databases between 2005 and 2019. Forty-four patients with a fibro-osseous lesion and complete medical records were identified, representing an incidence rate of 3.64%. Cemento-osseous dysplasia (COD) was the most common group of FOLs present in our patient-cohort (*n* = 20, 45%), followed by ossifying fibroma (*n* = 17, 39%) and fibrous dysplasia (*n* = 7, 16%). They were no cases of familial gigantiform cementoma or osteochondroma. Females were predominantly affected (73%) compared to males (27%) and the mean age of the patients at the time of diagnosis was 40.54 ± 13.66 years. Most lesions were located in the mandible (86%). The diagnostic imaging technique of choice was CBCT (68%), followed by PAN (18%)**,** and both imaging modalities were used in combination in 9% of patients. CT scans were used in only 5% of patients. In most patients (95%), a biopsy was performed after radiological examination as an additional diagnostic tool. After initial histopathologic diagnosis, resective surgery was performed in 73% of patients. Most patients were recalled for postoperative follow-up examination every six months (58%) and 23% every 12 months. Some patients were instructed to make an appointment for a follow-up examination if requested (19%). During follow-up, no malignant transformation of these lesions was observed in our patient cohort (Figure 1, Table 1).

### 3.2. Parameter Comparison: Cemento-Osseous Dysplasia, Ossifying Fibroma and Fibrous Dysplasia

**Figure 2:**
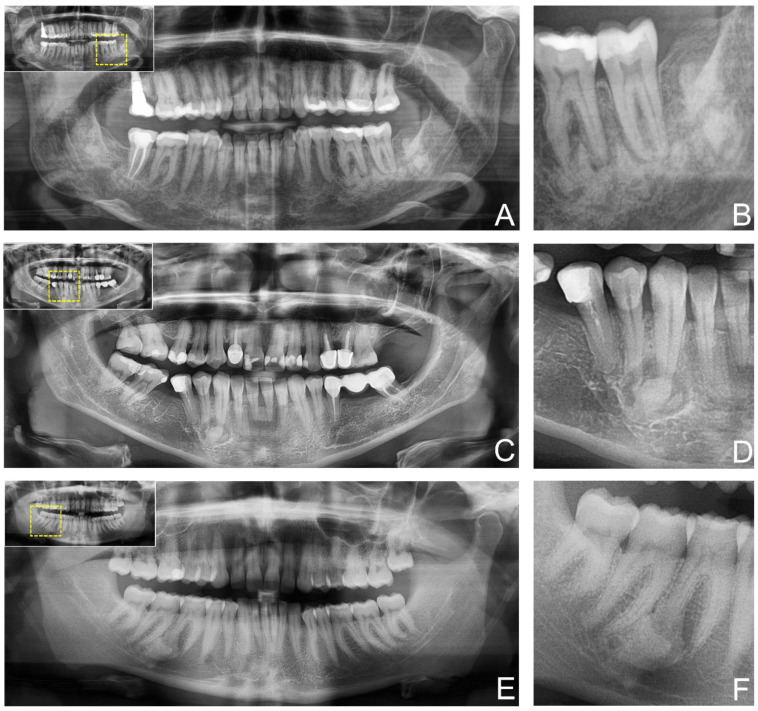
Study participants’ two-dimensional panoramic radiography (PAN) providing anatomical information of the fibro-osseous lesion. (**A**) shows an ossifying fibroma in the posterior mandible. In contrast (**C**) shows cemento-osseous dysplasia at the canine in the fourth quadrant, while (**E**) shows a cemento-osseous dysplasia at the first molar in the fourth quadrant. For orientation, the dotted rectangles in the corner show the enlarged area of the region of interest (**B**,**D**,**F**).

Peri-apical cemento-osseous dysplasia was the most frequent subgroup observed in our patient cohort (30%), closely followed by focal cemento-osseous dysplasia (focCOD) (25%). The least common was the florid type (FCOD) (5%). However, in most cases (40%), no information regarding the COD subtype was found in the patient-files. In this cohort, no further classification of the different subtypes of ossifying fibroma (cemento-ossifying, juvenile trabecular and juvenile psammomatoid ossifying fibromas) as well as fibrous dysplasia (polyostotic and monostotic forms) was reported. A clear gender predilection was observed with cemento-osseous dysplasia (COD), with 95% of patients being female, whereas for ossifying fibroma and fibrous dysplasia, there were only slightly more females affected, respectively 47% and 57%. Cemento-osseous dysplasia and ossifying fibroma were diagnosed in the fourth decade of life, with a mean age of 42.85 ± 13.45 years and 43.70 ± 11.84 years respectively. In contrast, fibrous dysplasia occurred mostly in the second decade of life with a mean age of 27.42 ± 12.02 years. For cemento-osseous dysplasia and ossifying fibroma, most lesions were localized in the mandible. However, for fibrous dysplasia, the maxilla was more frequently affected (57%). For all three groups of FOLs, CBCT imaging was the preferred radiological examination tool. CT scans were rarely used in cemento-osseous dysplasia and ossifying fibroma but were performed in 29% of the cases of fibrous dysplasia. Regarding radiological features, cemento-osseous dysplasia and ossifying fibroma presented predominantly a mixed image (65% and 53% respectively), showing both radiolucent and radiopaque areas. Fibrous dysplasia showed mixed and completely radiopaque images with the same frequencies (43%). Cemento-osseous dysplasia and ossifying fibroma most often presented with well demarcated borders on radiographic images, whereas fibrous dysplasia was predominantly poorly defined (71%) (Figure 2).

Bone biopsies for histopathological diagnosis were taken in 100% of patients with ossifying fibroma and fibrous dysplasia; slightly less patients with cemento-osseous dysplasia underwent a biopsy (90%). Surgical resection of the lesion was mostly performed in patients with ossifying fibroma (41%), followed by fibrous dysplasia (29%) and cemento-osseous dysplasia (15%). The main complaints before the surgical intervention were pain, swelling, or sensory disturbance; otherwise, the occurrence of a relapse was an indication. A recurrence of cemento-osseous dysplasia in 5% and ossifying fibroma in 6% was observed after initial resection. The postoperative follow-up interval for cemento-osseous dysplasia was set at 6 months in 45% of cases, while for the rest, the follow-up interval was largely determined according to the patient’s needs. For ossifying fibromas, the interval was set at 6 months in 76% of cases, whereas for fibrous dysplasia, the follow-up interval was set at both 6 months and 12 months in approximately 50% of cases (Table 2 and Table 3).

## 4. Discussion

FOLs are a rare, heterogeneous, and challenging group of oral and maxillofacial pathologic lesions whose etiopathogenesis is generally unclear. Despite similar histopathological characteristics, each subtype’s demographic, clinical, and radiologic features are unique and essential for accurate diagnosis and proper treatment. However, only a few retrospective studies have addressed this topic and there is no consensus on treatment and follow-up protocols in the current literature. Therefore, this retrospective study aimed to evaluate the incidence of FOLs and to reassess the efficacy of case-specific treatment management from a clinical, radiological, and histopathological perspective based on 14 years of data in a Swiss patient-cohort. 

From a demographic perspective, the actual prevalence of FOL in general and its subtypes is unknown and can only be estimated, as most of the reports performed to date depend on the source of the data, whether its origin was from oral pathology laboratories or oral and maxillofacial services. The studies with the largest patient cohorts estimated the most common occurrence of a subtype of FOL as ossifying fibroma at 77% and fibrous dysplasia at 23% in a patient cohort from China with 127 participants [19], 51% and 43% in a cohort of 122 cases in Thailand [20], while a study from Jamaica showed an occurrence of 31% and 47%, respectively [21]. These three retrospective studies used only data from oral pathology databases or only lesions treated with surgery. However, a Brazilian cohort study of 143 patients, which additionally focused on diagnostic imaging in the oral and maxillofacial regions, found 69% cemento-osseous dysplasia and approximately 15% each of osseous fibromas and fibrous dysplasia [2]. Therefore, the results obtained in this study (Cemento-osseous dysplasia (COD) (45%), followed by ossifying fibroma (39%) and fibrous dysplasia (16%)), should be considered with caution and put in the context of the data available in the literature. 

**Figure 3:**
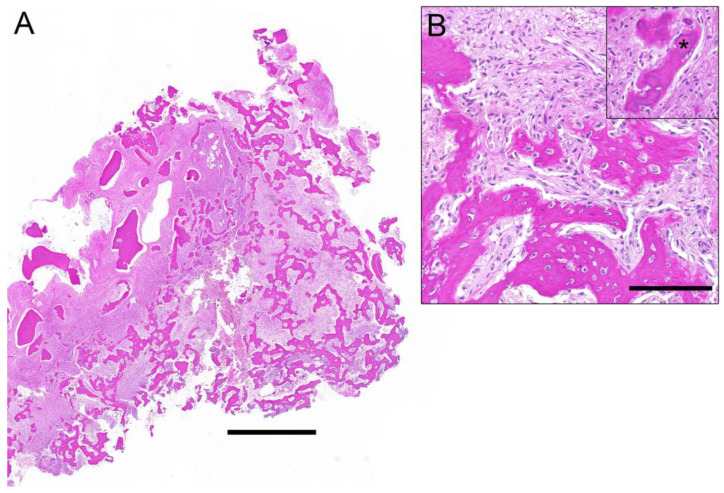
Histological overview of a fibro-osseous lesion (**A**) depicts irregular bony trabeculae with different grades of maturation and a fibroblastic spindle cell proliferation in between. The magnification (**B**) shows the bony islands with lack of osteoblastic rimming and the interjacent bland spindle cell proliferation. Inset illustrates the small spheroid cementum-like particles (asterisk). Correlation with radiological images rendered a diagnosis of cemento-osseous dysplasia. Scale bar 1 mm (**A**) and 100 µm (**B**).

Cemento-osseous dysplasia, the most common fibro-osseous lesion, is thought to be a non-neoplastic and reactive process in the tooth-bearing area with a predilection for the mandible in African and African American middle-aged women, whose etiopathogenesis possibly originates from the periodontal ligament [22]. The results of this study confirm the literature, as 95% of the affected patients were women with a lesion localized mainly in the mandible. In general, three subcategories can be distinguished: periapical cemento-osseous dysplasia affecting the periapical region of the anterior mandibular teeth, focal cemento-osseous dysplasia which concerns a single tooth often in edentulous areas, and florid cemento-osseous dysplasia affecting multiple quadrants [22]. From a radiological perspective, the appearance is specific to each subtype and may be focal or multifocal. The border of the lesion is usually well-defined and sclerotic, with an inner central area of radiopacity and an outer irregular area of radiolucency [23]. However, the findings of the present study show greater diversity in radiological appearance of cemento-osseous lesions. Depending on the stage, lesions may show a complete radiolucent, mixed, or radiopaque image [2], which were all three observed in our cohort. Histopathologically, the specimen is hemorrhagic, brown, and has granular fragments [24]. Although the three subtypes of cemento-osseous dysplasia differ in clinical and radiologic expression, they share the same microscopic features. Thereby most lesions have a fibrous stroma with loose fibroblasts and collagen with mineralized curved trabeculae of woven bone and cementum-like material and are well vascularized, with lesions becoming denser and less cellular as they mature [22] (Figure 3). 

**Figure 4:**
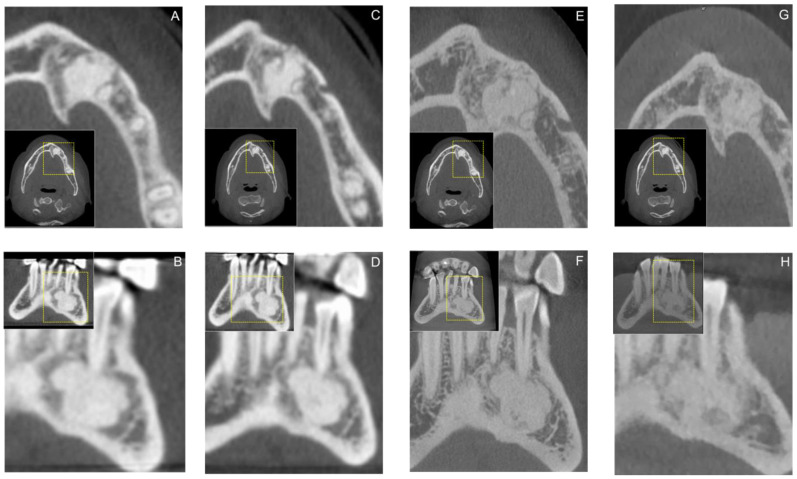
Axial (**A**,**C**,**E**) and Coronal (**B**,**D**,**F**) reconstructions of a study participants cone-beam computed tomography (CBCT) demonstrating annual radiological follow-up of cemento-osseous dysplasia. Additionally, the most recent follow-up on axial (**G**) and coronal (**H**) CBCT reconstructions of the region of interest using a low-dose imaging protocol is shown. For orientation, the dotted rectangles in the corner show the enlarged area of the region of interest.

Bone biopsies for histopathological examination are often deemed unnecessary. Nevertheless, in the present study, biopsies were performed in most cases, probably due the frequent atypical or unspecific radiologic presentation of the lesions, requiring histopathological analysis as an additional diagnostic modality to rule out other possible bone lesions of benign or malignant origin, presenting with similar clinical and radiological characteristics. In general, cemento-osseous dysplasia does not require treatment, as routine radiological follow-up is the therapeutic modality of choice (Figure 4). However, in cases of florid cemento-osseous dysplasia, symptoms might occur, and osteomyelitis may develop, which can require invasive surgical treatment [25].

Ossifying fibromas have a neoplastic potential, with some neoplasms exhibiting substantial growth potential [22]. It is usually diagnosed in the third to fourth decade of life, with a female predilection (5:1) and preferential occurrence in the posterior region of the mandible [19,20,21]. With a mean age at diagnosis of 43.70 ± 11.84, a female predilection (47%) and a predominantly mandibular localization (88%), the results of this study support the above-mentioned findings of the literature. Nevertheless, it is unclear whether geographical and ethnical factors may have an influence on these observations, as differences were discovered between Asian and African patient cohorts [26]. Clinically, this pathology most commonly manifests as painless bone swelling, with smaller tumors often being asymptomatic and often occurring as incidental findings on radiographs, with both factors confirmed by the results of this study [22,27,28]. The juvenile variants (JTOF and JPOF), which have no sex-specific predilection and affect most patients in their second decade of life, are characterized by rapid growth and expansion, particularly affecting the maxilla and maxillary sinus, leading to visual changes and dysfunction of the maxillary sinus [17,22]. From a radiological perspective, they have an oval or round shape with mixed-density appearances, but radiolucent cases may account for up to 20% of cases, consistent with the findings observed in this study [29]. Due to encapsulation by fibrous tissue and separation from the cortical bone, they are radiologically characterized by a well-defined radiopaque margin and have a low recurrence rate after surgical treatment [17,30]. Depending on the state of maturity of the lesion and the quantity of mineralized substance, the inner density may be variable, and a sclerotic cortical line may be apparent at the interface with the surrounding osseous tissue [26]. Displacement of adjacent teeth or root resorption can be observed in ossifying fibromas. However, the juvenile variants exhibiting a more aggressive growth pattern can also affect the brain [31]. Macroscopically, the specimen collected for biopsy is often yellow-white and of granular consistency [24]. Histopathological features are hypercellularity of the fibrous tissue, which is usually distributed in a storiform pattern with incorporated trabeculae of woven and lamellar bone or spherules of cementum-like material [29]. Due to the true neoplastic origin of the lesion, a biopsy for diagnostic purposes and subsequent complete surgical excision is recommended [2]. In our patient cohort, ossifying fibroma was biopsied in 100% of patients following initial radiographic evaluation, underlining the importance of histopathological examination as a key diagnostic tool. In contrast, a complete resection of ossifying fibromas was performed less frequently in our cohort, probably since the lesions often presented as asymptomatic and did not show an aggressive growth tendency. 

**Figure 5:**
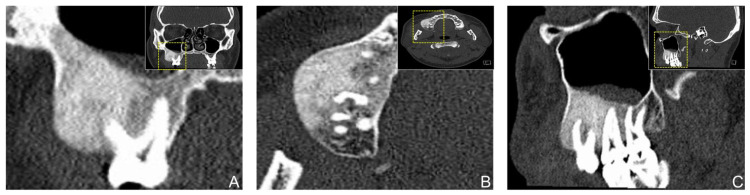
Coronal (**A**), axial (**B**), and sagittal (**C**) reconstruction of a study participant’s computed tomography (CT) showing a fibrous dysplasia in the posterior maxilla. Clinically, the patient presented with swelling associated with tenderness and hypoesthesia.

Fibrous dysplasia represents a skeletal disorder of the osseous tissue with a predominance of the humerus, femur, ribs, and bones of the skull. The monostotic form of the disease affects only one single bone and is the predominant type (70–80%), whereas the polyostotic form affects multiple bones, is associated with hormonal disturbances and skin changes, and often occurs in association with McCune-Albright syndrome [22,29]. The pathogenesis of fibrous dysplasia is associated with GNAS gene mutations, resulting in dysregulation and overproduction of cAMP, which alters the cellular properties of bone osteoprogenitor cells and leads to abnormal bone development [22]. It is usually diagnosed in affected patients’ first to the third decade of life, with no gender or race predilection [19,21,29,32]. Mostly gnathic bones may be affected by mild to severe mass expansion, which affects the adjacent anatomical structures and occurs predominantly unilaterally and in the maxilla [22]. Intraoperatively the area affected by expansion is not clearly distinguishable from the adjacent healthy bone tissue [17]. The results obtained in this study confirm the trends identified in previous reports [21,29,33], as no clear gender predilection was identified and the lesions were initially diagnosed in the third decade of life (mean age 27.42 ± 12.02 years), with the maxilla being predominantly affected (57%). Nevertheless, other investigations reported a similar extent of mandibular and maxillary involvement [19,34]. Radiologically, most cases of fibrous dysplasia show a characteristic ground-glass appearance and poorly defined peripheral border on mixed and radiopaque x-rays [22,29]. Immature areas were mostly radiolucent, whereas more mature lesions appeared more sclerotic [35]. The findings of this study confirm the predominantly poorly defined radiological presentation of the lesions (71%) (Figure 5). 

Macroscopically, the specimen collected for biopsy is often grayish-white in color and has a rubbery and compressible texture. Histology, often characterized by the terms “alphabet soup” or “Chinese character”, reveals predominantly a cellular fibrous tissue composed of fibroblasts and collagen, with mineralized curved trabeculae of woven bone [33,36]. From a pathology-oriented perspective, the results of this study support the statement that biopsy is essential for the diagnosis of fibrous dysplasia, as they were performed in all patients with this type of lesion. Histopathological analysis is not only essential for discovering a potential malignant transformation of the lesions, which has been reported in previous literature [18], but also to initiate an adequate subsequent treatment that can range from invasive surgery to follow-up or bisphosphonate therapy for the polyostotic form [32]. 

The rarity of fibro-osseous lesions in this demographic region might explain the relatively low number of patients included in the present cohort, compared to studies performed in other countries. Therefore, the results obtained, although mostly in line with current literature findings, are of low statistical value, which constitutes a limitation of the present study. However, the sample size does not allow for generally valid conclusions. Further studies with larger cohorts are needed to confirm the identified trends with higher reliability and validity, allowing ideal sample size calculation. In addition, conducting a regression analysis to investigate a possible cause-effect relationship could be interesting, as it is essential to consider possible confounding variables. Nevertheless, the following recommendations can be proposed to optimize and modernize decision making in clinical and radiological workflows: The recent introduction of digitally guided bone biopsies has greatly reduced the risk of damage to adjacent anatomical structures and the overall invasiveness of the procedure [37,38]. As a result, bone biopsies have become more common in recent years, which could lead to an increase in the effective number of histopathologic reports of FOLs. In addition, recent advancements in biomedical imaging have enabled the use of low-dose CBCT protocols in clinical practice with similar radiation exposure to conventional imaging techniques to detect bony lesions in the jaw without diagnostic restrictions [39,40]. By providing three-dimensional information about the region of interest, these imaging protocols can be implemented into future radiology workflows as an introductory imaging modality and postoperative follow-up, leading to a higher detection rate of incidental findings of FOLs and thus improving patient outcomes. However, these findings should serve as a reminder of the truly neoplastic nature of many odontogenic and non-odontogenic jaw lesions. A “wait and see” strategy can lead to growth of these lesions, which, although asymptomatic, can spread over a large area and cause significant surgical rehabilitation deficits despite minimally invasive surgical removal. For this reason, a multimodal therapeutic approach should always be considered in the treatment of FOLs. 

## 5. Conclusions

An interdisciplinary approach, analyzing all case-specific factors, is elementary to enable an accurate diagnosis and thus appropriate personalized treatment planning for benign fibro-osseous lesions. In this context, demographic data, medical history, intraoperative findings, and, most importantly, histopathological, and radiological features are essential for an accurate diagnosis. To provide appropriate case management and thus therapeutic approaches with improved risk-benefit ratios, the combination of pathological and radiological examination will continue to provide the most accurate and definitive diagnosis. However, considering the comprehensive clinical, imaging, and histopathological analysis of the patient’s disease is key to avoiding inappropriate treatment and involves postoperative follow-up imaging recommendations.

## Figures and Tables

**Table 1:** Overview: Demographic, clinical, radiological, and pathological parameters for all fibro-osseous lesions.

Parameter	Category	Result	Percentage
FOL group distribution	Cemento-osseous dysplasia Peri-apical COD FocCOD ^1^ FCOD ^2^ No information Ossifying fibroma Fibrous dysplasia Familial gigantiform cementoma Osteochondroma	20 6 5 1 8 17 7 0 0	45% 30% 25% 5% 40% 39% 16% 0% 0%
Age		40.54 (SD:13.66)	
Gender	Female Male	32 12	73% 27%
Localization	Mandible Maxilla	38 6	86% 14%
Imaging Technique	CBCT OPT CBCT/PAN CT	30 4 2	68% 18% 9% 5%
Biopsy	Yes Symptoms No symptoms No	42 12 30 2	95% 29% 71% %
Resection surgery	Yes No	12 32	27% 73%
Follow-up interval (every *n* months)	6 months 12 months 24 months If requested No information	25 10 0 8 1	57% 23% 0% 18% 2%
Postoperative complications	Malignant transformation Reccurrence of lesion Expansion of lesion Symptoms	0 2 0 0	0% 5% 0% 0%

^1^ FocCOD = focal cemento-osseous dysplasia, ^2^ FCOD = florid cemento-osseous dysplasia.

**Table 2:** Parameters for each type of fibro-osseous lesion.

Parameter	Category	COD ^1^	OF ^2^	FD ^3^
Age		42.85 (SD:13.45)	43.70 (SD:11.84)	27.42 (SD:12.02)
Gender	Female Male	19 (95%) 1 (5%)	8 (47%) 9 (53%)	4 (57%) 3 (43%)
Localization	Mandible Maxilla	20 (100%) 0	15 (88%) 2 (12%)	3 (43%) 4 (57%)
Imaging Technique	CBCT OPT CBCT/PAN CT	17 (85%) 0 (0%) 3 (15%) 0 (0%)	9 (53%) 7 (41%) 1 (6%) 0 (0%)	4 (57%) 1 (14%) 0 (0%) 2 (29%)
Biopsy	Yes No	18 (90%) (10%)	17 (100%) 0 (0%)	7 (100%) 0 (0%)
Resection surgery	Yes No	3 (15%) 17 (85%)	7 (41%) 10 (59%)	2 (29%) 5 (71%)
Follow-up interval (every *n* months)	6 months 12 months 24 months If requested	9 (45%) 5 (25%) 0 (0%) 6 (30%)	13 (76%) 3 (18%) 0 (0%) 1 (6%)	3 (50%) 3 (50%) 0 (0%) 0 (0%)
Postoperative complications	Malignant transformation Recurrence of lesion Expansion of lesion Symptoms	0 (0%) 1 (5%) 0 (0%) 0 (0%)	0 (0%) 1 (6%) 0 (0%) 0 (0%)	0 (0%) 0 (0%) 0 (0%) 0 (0%)

^1^ COD = cemento-osseous dysplasia, ^2^ OF = ossifying fibroma, ^3^ FD = fibrous dysplasia.

**Table 3:** Distribution of radiological features (*n*, %) for each fibro-osseous lesion subtype.

Parameter	COD ^1^	OF ^2^	FD ^3^
Type of image Radiolucent Mixed Radiopaque	2(10%) 13 (65%) 5 (25%)	6 (35%) 9 (53%) 2 (12%)	1 (14%) 3 (43%) 3 (43%)
Limits of lesion Well defined Poorly defined	20 (100%) 0 (0%)	16 (94%) 1 (6%)	2 (29%) 5 (71%)

^1^ COD = cemento-osseous dysplasia, ^2^ OF = ossifying fibroma, ^3^ FD = fibrous dysplasia.

## Data Availability

The data presented in this study are available on request from the corresponding author. The data are not publicly available due to privacy restrictions.

## Abbreviations

COD	cemento-osseous dysplasia
COF	cemento-osseous fibroma
FD	fibrous dysplasia
FGC	familial gigantiform cementoma
JPOF	juvenile psammomatoid ossifying fibroma
JTOF	juvenile trabecular ossifying fibroma
